# We are still in it: a conceptual model for moral injury and burnout in alternative response programs to guide intervention

**DOI:** 10.3389/fpsyt.2026.1735265

**Published:** 2026-03-02

**Authors:** Miranda Worthen, Soma De Bourbon, Omar Breedlove, Courtney Ewing, Joseph Graterol, Amelia Harmon, Nakia Hines, Erik Lundgren, Michael Mason, Michael Marchiselli, Vania Mendoza, Khoa Nguyen, Britt Rubin, April Sloan, Shira Maguen

**Affiliations:** 1 Department of Public Health, San José State University, San José, CA, United States; 2 Department of Sociology and Interdisciplinary Social Sciences, San José State University, San José, CA, United States; 3 Richmond Area Multi-Services (RAMS), Inc., San Francisco, CA, United States; 4 San Francisco Fire Department, San Francisco, CA, United States; 5 Department of Emergency Medicine, School of Medicine, University of California, San Francisco, San Francisco, CA, United States; 6 Mental Health Service, San Francisco VA Health Care System, San Francisco, CA, United States; 7 Department of Psychiatry and Behavioral Sciences, School of Medicine, University of California, San Francisco, San Francisco, CA, United States

**Keywords:** burnout, emergency medical technicians (EMTs), moral distress, moral injury, paramedics, participatory action research (PAR), peer support specialists

## Abstract

**Introduction:**

About 20% of police calls involve a person experiencing a behavioral health crisis. To better address these community member’s needs and reduce risk of harm, jurisdictions are developing programs that shift calls for crises related to homelessness, addiction, and mental health to non-law enforcement alternatives. In responding to these calls, first responders working in these alternative response programs are exposed to a different set of moral dilemmas and pressures, increasing their risk of developing moral injury.

**Methods:**

The Alternative Response Program Moral Injury and Burnout Model highlights causes of exposure to potentially morally injurious events and the interplay between moral distress, moral injury, and burnout among first responders working in alternative response programs. The conceptual model was developed through a multi-year participatory action research (PAR) study with members of a large urban alternative response program. The article draws on a highly diverse set of data sources that contributed to the development and refinement of the model, including focus group discussions, analysis of call logs, assessment of moral injury, and reflective conversations with members and leaders of the alternative response program. Through our collaboration, we identified contributory factors that can produce moral injury and opportunities for interventions to mitigate moral injury. We piloted several interventions collaboratively developed with team members and hypothesized to reduce moral distress and injury based on this model.

**Results:**

Our model explains sources of moral dilemmas and how these can lead to moral distress, moral injury, and burnout. The model also points to intervention pathways to reduce or prevent these outcomes. We provide illustrations of how moral injury arises, using data from the program members, describe six pathways, and share examples for how moral injury may be mitigated.

**Discussion:**

Despite scarce research in first responders, moral injury and burnout are growing concerns in alternative response programs. Our goal was to amplify participants’ voices to highlight both their struggles as well as proposed interventions fostering resilience. Through close collaboration, we developed a moral injury model that resonates for these first responders and can guide a better understanding of issues and pathways for interventions among high-risk occupational groups.

## Introduction

Moral injury is conceptualized as the biopsychosocial, behavioral, and spiritual sequalae that may arise from events in which someone acts in a manner that goes against their deeply held morals or values, or from failing to prevent or witnessing such events (1, 2). There is a growing literature on moral injury for those working in high-risk occupations, such as veterans and healthcare workers (1, 3, 4). While there is limited empirical research on first responders, several articles discuss the importance of the moral injury construct among diverse first responders, including paramedics and emergency medical technicians (EMTs) (5–10). In a recent article, Litz (3) described the need for the field to build and strengthen moral injury theory to support hypothesis generation, research on the etiology of moral injury, and interventions to mitigate moral injury.

We present the Alternative Response Program Moral Injury and Burnout Model, a conceptual model highlighting causes of exposure to potentially morally injurious events (PMIEs) and the interplay between moral distress, moral injury, and burnout among first responders working in the emerging field of non-law enforcement alternative first response. This conceptual model was developed through a multi-year participatory action research study in a large urban alternative response program in northern California.

### Alternative response programs

Nationally, an estimated 20% of police calls involve a person experiencing a mental health or substance use crisis (11). While estimates vary, it is thought that 25% – 50% of all people killed by police officers are experiencing this type of crisis (12). As jurisdictions seek better strategies to address the needs of people experiencing mental health or substance use crises while reducing potential harm and respecting autonomy, many are developing multidisciplinary alternative response programs that do not involve law enforcement. Alternative Response Programs differ from co-response models, where a behavioral health specialist may accompany police to respond to a call, and may also be called Alternative Crisis Response Programs, Alternative First Response Programs, or Alternative Emergency Response Programs.

Alternative response program composition and dispatch models vary widely. Some programs have dedicated phone referral lines, respond during limited hours, and are staffed by trained community responders who can work with clients to identify social needs and refer and transport to agencies (e.g. Mobile Assistance Community Responders of Oakland (13) and Atlanta’s Policing Alternatives and Diversion Initiative (14). Other programs are dispatched through 911, have trained paramedics or EMTs and/or behavioral health clinicians, and respond to calls 24/7 (e.g. Rochester’s Person in Crisis Team (15) and Denver’s Support Team Assisted Response (16)). Many programs also include peer support specialists, behavioral health workers who have lived experience with homelessness, mental illness, and/or substance use who provide peer-based counseling services and support (e.g. Durham’s Holistic Empathetic Assistance Response Team (17) and Arlington, Virginia’s Mobile Outreach Support Team (18)).

In each jurisdiction that uses 911-dispatched alternative response programs, the specific types of calls that these teams respond to may vary. For example, many programs respond to calls for “wellness checks” or reports of a person experiencing a non-violent mental health crisis. Programs with paramedic first responders may also respond to calls for drug overdose. In responding to these new types of calls (e.g., behavioral health crises, wellness checks) that are not for medical emergencies requiring transport to a hospital, dispatched first responders working in these non-law enforcement crisis response programs are exposed to an entirely different set of moral dilemmas and pressures than encountered in traditional emergency medical services. For example, many unhoused clients experiencing substance use disorder decline offers of shelter or treatment. Although these engagements may support clients emotionally and foster increased trust in the system of care, the client is left in the community without improvement in their material circumstances, which can be distressing for some first responders. These exposures can put alternative response workers at risk of moral injury, which can not only impact the alternative response workers, but impedes their ability to effectively serve clients and can create structural challenges for municipalities, as moral injury can be associated with burnout and attrition (19).

Given the importance of having a framework to better understand how these work experiences impact first responders to better guide leadership support, interventions, and policy, this article presents a conceptual model developed through a four-year participatory action research (PAR) study with paramedics, EMTs, and peer support specialists working in non-law enforcement 911-dispatched alternative response programs in a large urban setting. We will describe the PAR approach and explain the research process used to develop and validate the conceptual model. We will then present the Alternative Response Program Moral Injury and Burnout Model and share the qualitative data that elaborate, explain, and support the model. We will also identify hypothesized and piloted critical interventions to mitigate and prevent moral distress, injury, and burnout based on the conceptual model and qualitative data. Finally, we will discuss how the model fits with existing models of moral injury and extends our understanding of moral injury in high-risk occupations. In keeping with the PAR approach, this manuscript was co-developed and is co-authored by program collaborators and academic partners.

## Methods

### Participatory action research

This study employed an approach to research called participatory action research (PAR). PAR is an approach to research that values academic, experiential, and technical knowledge, engaging in a partnered process where decisions are made collectively about what problems deserve focused attention and what solutions may serve to mitigate these problems (20). Cornwall and Jewkes have argued that the primary difference between traditional research and PAR “lies not in methods, but in the attitudes of researchers, which in turn determine how, by and for whom research is conceptualized and conducted” (20, p. 1667). Baum, MacDougall, and Smith describe PAR as “collective, self-reflective inquiry that researchers and participants undertake, so they can understand and improve upon the practices in which they participate and the situations in which they find themselves” (21). In this study, we employed a cyclic process of problem identification, participatory research, intervention development, implementation, and evaluation aiming to create meaningful social change while producing generalizable knowledge. Consequently, intervention development, pilot testing, and evaluation occurs within the framework of the PAR cyclical model rather than a standalone experimental intervention.

### Setting

The setting for this study was an alternative response program in a large city in northern California. The alternative response program was launched in 2020 and is run through the Fire Department, in collaboration with local public health and emergency management departments. The study began in 2021, less than a year after the alternative response program began, and at first the driving question behind the collaboration was to try and answer the question “How can this alternative response team work to improve the health and wellbeing of our city?” As the academic research partners and response team leadership in the Fire Department met and began to discuss the experiences of first responders in the program, we collaboratively identified moral injury as one of the challenges likely facing program personnel as they engaged in their work. From 2022 – 2025, one of the primary foci of the study was on understanding and mitigating moral injury among members of the alternative response program.

While team composition has varied, during most of the study period, the team included a paramedic member of the Fire Department and a peer support specialist who respond together to each 911 call. Teams have also included a licensed behavioral health clinician (e.g. social worker), an EMT (also a member of the Fire Department), or a Homeless Outreach Team member who can perform housing assessments and referral placements. Teams are dynamically deployed, meaning that for the duration of a shift, team members are either responding to a call or are at a designated spot in their vehicle ready to respond. The program has a high call volume, responding to over 50,000 calls between November 2020 and March 2025. Most calls are for non-violent behavioral health crises, though the teams also respond to overdoses and other problems facing community members experiencing homelessness (22).

### Data contributing to the conceptual model

The long term and strong partnership between the academic researchers, leadership of the program, and members of the response teams, as well as the cyclical nature of the PAR approach allowed for “methodological pluralism” (21), with a highly diverse set of data sources that contributed to the development and refinement of the conceptual model we present in this article. The conceptual model was developed through pairing the PAR approach with analysis using a modified constructivist grounded theory approach (23) and then, as described by Cole, returning to PAR partners to “test, revise, enrich, refine, and discuss what was emerging from the data” (24). The continual return to PAR partners “enabled testing for resonance, rigor, and applicability for practice” as the conceptual model was honed (24). **Table 1** describes each data source, the timing of data collection and analysis, and the purpose of each type of data. We will briefly elaborate on these data sources.

**Table 1:** Data sources contributing to conceptual model.

Data source	Timing	Description and general purpose	Specific contribution to conceptual model
Key personnel team meetings	Biweekly 2021 – 2024	Discussions between Department leadership in the alternative response program and academic partners to identify challenges, collaboratively develop interventions and problem-solve, assess progress, provide feedback, and promote co-learning.	Discussions were informal and formal opportunities to share examples and insights about moral injury from department leadership to academics and from academics to department leadership. These conversations contributed to the initial development of the model, as well as member checking.
Formative focus group discussions	7 times between 2022 – 2023	Focus group discussions with Department members and peer support specialists working on the alternative response team about their work and moral injury.	Participants responded to a question about how they thought their work might contribute to moral injury and what practices might prevent or reduce moral injury. Data were initially thematically analyzed and shared back with leadership and members for participant validation. Repeated analysis using a constructivist grounded theory approach supported the initial development of the model.
Encounter log data analysis	1 time in 2022	Review of the narrative description of the encounters for approximately 10,000 calls responded to by the alternative response program.	Members of the program described events that occurred during encounters, including potentially morally injurious exposures. These data contributed to the initial development of the model.
Peer support specialist advisory board meetings	Monthly 2023 - 2024	Discussions between peer specialists on the advisory board and academic partners to discuss the overall intervention, what peers bring to alternative response programs, and debrief challenges to working in a multidisciplinary alternative response team.	Many conversations surfaced experiences where peers were exposed to potentially morally injurious events and what approaches peers used to address these exposures, including workplace-level approaches. The moral injury conceptual model was presented in these meetings and participants provided feedback (co-construction through elaboration and participant validation).
Moral injury assessment and analysis	1 time in 2024	Assessment of Department members using the Moral Injury and Distress Scale (MIDS).	Academic partners quantitatively analyzed MIDS exposure and symptom data from 292 participants and qualitatively analyzed 124 written descriptions of potentially morally injurious event exposures provided by survey participants. MIDS data played a primarily confirmatory role in the model development.
Alternative response team peer conversations	16 times in 2024	Set of four topical 60 to 90-minute conversations facilitated by academic partners for members of the alternative response teams designed to intervene on moral injury.	Members were provided brief didactic presentations about exposure to potentially morally injurious events, experiences of moral injury, and strategies to promote resilience. Then, participants talked with each other about their own experiences and made connections between the material and their work. These conversations played a primarily confirmatory role in the model development.
Reflective focus group discussions	4 times in 2024	Evaluative focus groups with members of the alternative response teams to understand experiences and perspectives of participants in the intervention, identify any adverse sequelae, discuss opportunities to sustain workplace-level changes, and to provide feedback on the moral injury conceptual model.	Participants described their experiences with the peer conversations and other aspects of the moral injury intervention. The moral injury conceptual model was presented in these meetings and participants provided feedback (co-construction through elaboration and participant validation).
Department report-backs	2 times in 2023 3 times in 2025	Meetings where the academic partners shared findings from focus groups and the moral injury assessment with Department members and facilitated discussion about the results.	In the first two report-backs, academic partners presented the emerging understanding of moral injury to department leadership and members, who asked questions, provided experiential reflections, and weighed in on proposed interventions to consider to mitigate moral distress and injury. In the subsequent three report-backs, academic partners presented on the mixed-methods results of the moral injury assessment and engaged in discussion about exposures to potentially morally injurious events, the development of moral injury, and the relations between moral injury and other mental health outcomes. These meetings allowed for member checking.
External alternative response conversations	4 times in 2023 – 2024	Discussions between the academic partners and people who work in non-project affiliated California alternative response programs.	During these conversations, academic partners discussed our understanding of moral injury among members working in alternative response programs with leaders of programs external to the study program, including soliciting feedback on the moral injury conceptual model. These conversations provided evidence for validation of the model.

Throughout the study period, Fire Department leadership and academic partners met weekly or bi-weekly. In 2022 – 2023, we conducted six focus groups with EMTs and paramedics working in the program and one focus group with peer support specialists working in the program. During each focus group, we defined moral injury and asked participants whether they thought their work put them in circumstances where they might experience moral injury and whether there were any practices in their workplace that help protect them from moral injury. Each focus group was audio recorded, transcribed, and thematically analyzed by two researchers. Focus group participants made several suggestions for interventions that they thought might reduce their exposure to potentially morally injurious events and help to mitigate moral distress and injury. In this period, we also examined de-identified encounter log data from over 10,000 calls to the alternative response program, reviewing the narrative descriptions of each encounter. Narrative review was aimed at understanding the breadth of call experiences, including both explanations of what actually happened as well as latent content about the intensity of the call.

From the initial thematic analysis of focus group data and encounter log review, we began to construct meaning diagrammatically through memoing and to visually depict sources of moral injury identified through the focus groups. This initial model was shared with department leadership and, separately, with members of the response program in early 2023. During these report-backs, leadership and members asked questions, provided experiential reflections, and weighed in on proposed interventions to consider to mitigate moral distress and injury.

In light of the strong support from members for the focus on moral injury, we added a researcher with extensive expertise in moral injury to the academic team. With this strengthened partnership, we designed a pilot study to test a series of interventions that we – meaning the academic partners, department leadership, and response team members – hypothesized might help to understand, prevent, and reduce moral distress and injury among team members.

The initial focus group discussions suggested that experiences of peer support specialists in the programs were distinct from that of EMTs and paramedics, largely because the organizations they work for have different work cultures and policies. Their different training, professional mandates, skills, and experience mean that they play different roles on the response team (e.g. peer support specialists self-disclose as a tool to instill hope and resiliency when connecting to clients and centering their needs). In some cases, teams work very well together, recognizing the complementarity of these distinct roles and in other cases there is friction in the team because of a lack of shared perspective and appreciation for what each team member contributes to the team. In order to have a separate space where academic partners on the study could learn from the experiences of peer support specialists, we established a peer support specialist advisory board, which brought together peer specialists from multiple programs, including but not limited to the alternative response program, and met monthly throughout the study.

In keeping with the PAR approach, we developed a series of efforts aimed simultaneously at understanding and intervening to improve moral distress, injury, and burnout for all members of the alternative response program. We will discuss some of these pilot interventions in more detail below. In brief, in 2024, with the support of the Fire Department, we conducted a department-wide assessment of moral distress and injury with a survey. While survey data were not central to the development of the conceptual model, they were used to validate the model as the survey was one of the few fully anonymous methods used during the study. Both the quantitative and qualitative results of the study have been previously published (7, 10). We then held a series of alternative response team conversations with team members (i.e. EMTs, paramedics, peer support specialists, and Homeless Outreach Team members). We held these conversations so that each shift had five sessions over five weeks. The conversations were aimed at both providing shared language and understanding around the impact of their everyday witnessing (25), moral injury and stress and also giving space to grapple with the ethical aspects of their work such as understanding their client population, issues around gender and race, and the long-term impacts of adverse childhood experiences. We also piloted holding weekly meals, where teams could come back to the station during their shift and eat a meal together, sometimes cooked by their supervisor and sometimes cooked by a team that was not in service. After these pilot interventions had been going for some time, we held reflective focus groups where we asked team members what they thought of the peer conversations and team meals.

During the study we had several opportunities to report our findings back to leadership of the Fire Department and the peer support specialist agency. We also reported findings back to the alternative response program membership. We connected with leaders of alternative response programs throughout California to share what we were learning about moral injury and hear more about the contexts in which other programs operated in order to try and identify common factors across programs.

## Conceptual model

**Figure 1:**
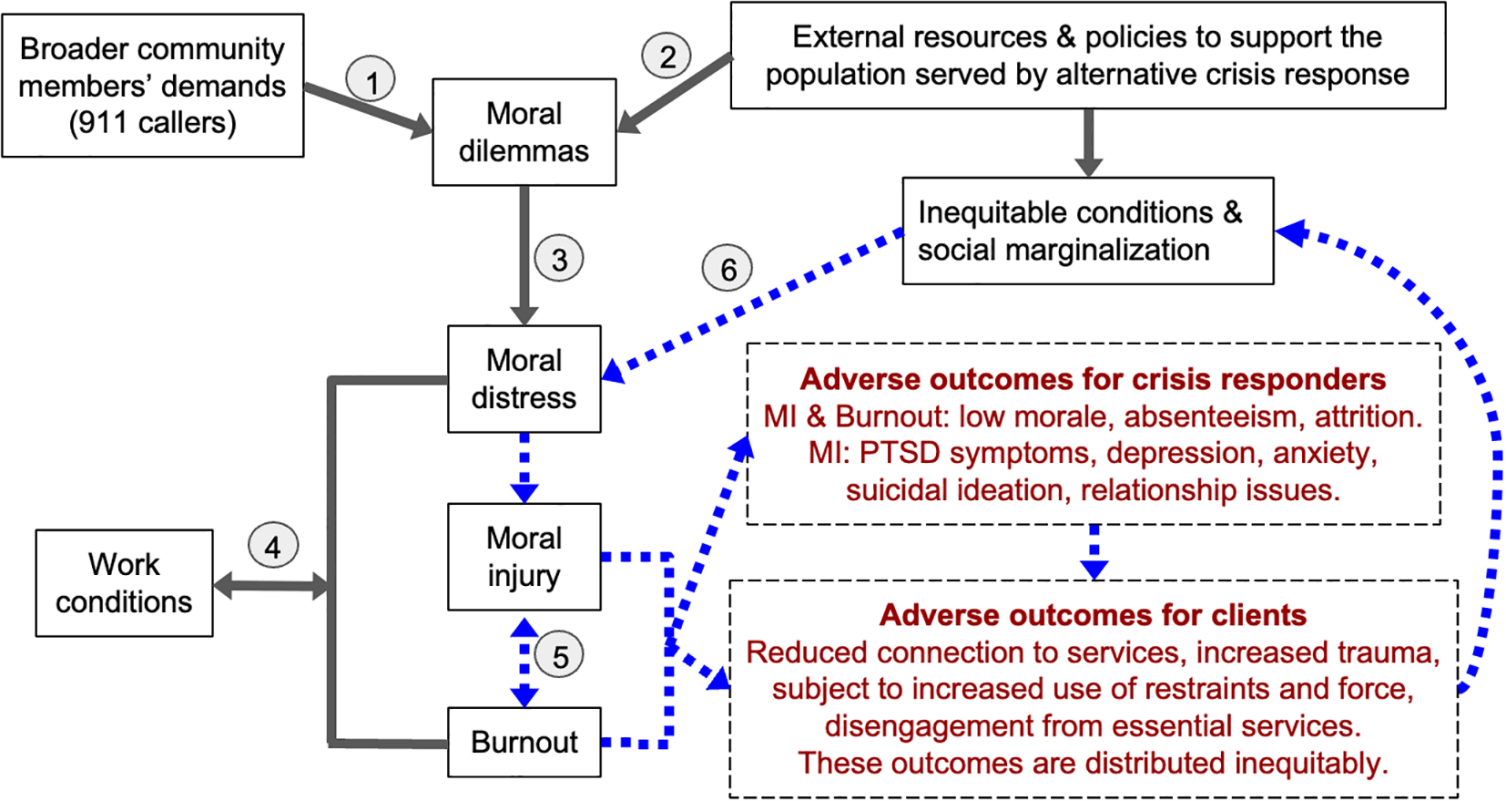
Alternative response program moral Injury and burnout model. The alternative response program moral injury and burnout model describes sources of moral dilemmas, which can lead to moral distress, moral injury, and burnout. The model posits that work conditions can contribute to and also protect against the development of moral distress and moral injury, which we conceptualize as on a continuum, and illustrates the interplay between moral injury and burnout. The model depicts adverse sequelae of moral injury and burnout for clients of the alternative response programs and for people working in the alternative response team (noted in dark red – dashed box). The model also identifies a feedback loop that amplifies these problems (noted in blue – dotted arrows)

The conceptual model explains sources of moral dilemmas and how these can lead to moral distress, moral injury, and burnout and also points to intervention pathways in order to reduce or prevent these outcomes (**Figure 1**). While moral distress, injury, and burnout have overlapping sources and symptoms, they each have unique components (**Table 2**). We conceptualize a moral dilemma as a situation that requires a difficult choice to be made, often involving conflict between values, which can lead to moral stress, low level normative stress resulting from the dilemma (26). The moral dilemma may also lead to moral distress, a more pervasive negative emotional experience related to the moral dilemma, without more prevalent impacts on functioning. Moral injury can result from persistent moral distress or repeated and/or severe exposure to PMIEs, causing impaired functioning and cognitions (2, 27). We conceptualize moral stress as on a continuum, with one end being normative, acute, and at times adaptive, and the other end resulting in persistent and severe moral injury symptoms (27). Burnout can result from chronic workplace stress and is defined by emotional exhaustion, depersonalization or cynicism, and a reduced sense of professional efficacy (28, 29). We also consider burnout as occurring on a continuum (29). While some symptoms of burnout and moral injury overlap (e.g. exhaustion or detachment) other symptoms are unique (e.g. hallmark symptoms of moral injury are feelings of guilt or shame and a sense of betrayal by leadership). Similarly, the sequalae of moral injury and burnout overlap (e.g. both may cause low morale or result in one’s decision to leave their position), with each also having unique sequelae (30, 31). Studies with healthcare workers have found that high degrees of moral injury and burnout are each associated with depression, suicidal ideation, and alcohol use, though burnout appears to be less strongly associated with these outcomes (27, 31).

**Table 2:** Definitions of constructs.

Construct	Definition
Moral dilemma	A situation that requires a difficult choice to be made, often involving conflict between values, which can lead to moral stress (26).
Moral stress	A low level normative stress resulting from a moral dilemma or stressor (26).
Moral distress	A pervasive negative emotional experience related to the moral dilemma, without more prevalent impacts on functioning. Moral distress is on a continuum of moral stress with one end being normative, acute, and at times adaptive, and the other end resulting in persistent and severe moral injury symptoms (27). Moral distress can arise from a single event or from a cumulative exposure to moral dilemmas or stressors.
Moral injury	The biopsychosocial, behavioral, and spiritual sequalae that may arise from events in which someone acts in a manner that goes against their deeply held morals or values, or from failing to prevent or witnessing such events (1, 2). Moral injury can result from persistent moral distress or repeated and/or severe exposure to potentially morally injurious events, causing impaired functioning and cognitions (2, 27).
Burnout	Burnout can result from chronic workplace stress and is defined by emotional exhaustion, depersonalization or cynicism, and a reduced sense of professional efficacy (28, 29). Burnout is distinct from moral distress and moral injury but these constructs overlap in their drivers and sequelae.

In our conceptual model, we hypothesize specific adverse sequela of moral injury and burnout for clients of the alternative response programs and for people working in the alternative response team (noted in dark red – dashed box). The model also identifies a feedback loop that amplifies these problems (noted in blue – dotted arrows).

The model operates at multiple levels or dimensions simultaneously. Arrows that show influences should not be considered literal pathways that suggest a temporal ordering, but rather contributory sources and factors. For example, an individual may be experiencing a high degree of moral distress from cumulative exposure to challenging circumstances (e.g. working with clients experiencing homelessness and substance use who are suffering but decline services), while also experiencing work conditions that diminish resilience to moral injury (e.g. a person may be required to take on a mandatory overtime shift or be assigned to work with an unfamiliar partner with whom the person has little trust). In this context, an experience of a PMIE caused by inadequate external resources may feel especially troubling, causing moral injury. Another example is that the team may encounter a disabled client who wants a shelter bed when none are available specifically for people who require a wheelchair, and they have to tell the client that they are unable to help, leaving the person on the street. In the context of cumulative exposure, exhaustion, and lack of trust in fellow team members, this particular exposure to a PMIE may cause moral injury symptoms.

In this section, we provide illustrations from this study of how moral injury may arise. For simplicity of illustration, we use the numbered pathways to anchor these examples, recognizing that oftentimes the pathways operate simultaneously, not sequentially, and from a societal level to an embodied one (32). After describing each pathway, we then provide examples for how moral injury may be prevented or mitigated.

### Pathway 1: broader community members’ demands can create moral dilemmas for alternative response teams

For dispatched alternative response programs, teams are mobilized in response to calls to 911. While typically, calls to 911 are for help for the calling party, for these alternative response programs, the caller is often reaching out about a behavior that the caller finds inappropriate or unwelcome and which they would like the responders to address. As one team member put it:

“Crisis is a big term … Is the crisis of the client? Or does the crisis lay on the neighborhood? Or on the society that is watching abnormal behavior in an individual? Because just we go on many, many calls, for example, where we have somebody calling 911. We get sent out there, and they don’t want people on the sidewalk. They’re like ‘they’re on the sidewalk, and they have all this stuff. They have their tent, and they’re blocking the sidewalk, and they’re yelling and screaming.’ And I tell them, ‘It’s their right … They have rights.’ They’re like, ‘Well, can’t you take them anywhere?’ Is what we often hear from the reporting party, and that becomes a teachable moment, because you need to educate the caller, the reporting party, that it’s unfortunate, we can work with this individual, but we can’t just scoop them up and kidnap them and take them somewhere. We just don’t do that.” (formative focus group discussions)

Another participant reflected:

“I am empathetic toward a lot of the homeowners and people that call and complain…. I get it. Like, I wouldn’t want to walk outside and have to have my small kids stepping over needles every day on my porch. I would be angry. But on the other hand, I understand that this [homeless] individual’s lived through a lot of trauma in their life that’s probably led to what’s going on … And there’s not resources that fit everybody, there’s not. And you know, we go into this really wanting to help the situation, and that fatigue of getting on scene and wanting to help everybody but not being able to, and that helpless feeling of, like I’m here. I want to help you. I don’t want to disappoint you.” (formative focus group discussions)

In the best of moments, team members are able to collaborate together, embracing their role in educating community members as well as supporting clients:

“A lot of the calls that we go on, we may have a client where we’re trying to de-escalate or assist. But we’re there probably more so for the reporting party … than we are for the actual client because there’s only so much we can do. So there’s a lot of quarter-backing where one of us is having, after the situation, whether you resolved it or not, having to talk to the community. And a lot of times, I think, it’s a little bit more positive if we actually have that discussion instead of just jumping in the van and leaving. But we’re not going to make everybody happy. And their expectations are, ‘I’m just going to call the Fire Department. It’s going to disappear.’ It’s not actually the case. There are just some things that we can’t do, there’s some things that we can’t fix. We don’t have enough resources. We need more resources. And that’s kind of a moral dilemma because we find ourselves all the time with somebody who wants something and we just don’t have. It’s frustrating.” (reflective focus group discussions)

### Pathway 2: external resources and policies to support the client population can create moral dilemmas for alternative response teams

Team members are directly responding to the demands of the community, oftentimes with inadequate resources to support the clients they are serving, thereby being immersed in ongoing moral dilemmas.

“…the broader community, they’re screaming at us. Like, ‘you guys don’t do anything. You guys are worthless. Taxpayers’ money go to waste.’ And we’re like, ‘well, if that’s how you feel, then you need to talk to your constituents and formulate policy,’ right?… We need some … entity to tell the community like, look, if you want things to change, we’re out here responding, we’re doing the best that we can do. We will respond 24/7, right? But the thing is, our hands are tied because of policies, resources, and the lack thereof of different places where we can take people. I mean, it’s terrible.” (reflective focus group discussions)

Another participant chimed in, saying:

“I think that’s frustrating, but we still come up with our Plan Bs, Plan Cs, and Plan Ds, and they’re not—they’re not great, but it’s a plan. And maybe we have to shove somebody in a corner alley with a bunch of blankets and come meet him the next day. That’s all we got.” (reflective focus group discussions)

There was a consistent challenge with insufficient resources, ranging from psychiatric hospital beds to safe shelter facilities. One paramedic compared the challenge of trying to do alternative response work without sufficient resources to what it would be like if an ambulance couldn’t rely on the hospital:

“I feel like if you translate it to an ambulance: The ambulance is looking at somebody in their house [having a heart attack] and saying, ‘hey, there’s going to be a cardiologist at the heart center. I’m going to take you there. They’re going to be open, they’re going to have a bed, and they’re going to do surgery.’ That person’s having a heart attack, so those are their needs. But we translate those to an unhoused client and say your needs are shelter, food, an ID, and some psych services. But I can’t guarantee … that anymore. And that’s where, you know, like we assume the hospital is open. If we showed up in the hospital and the doors are closed, people wouldn’t have that same trust in an ambulance … That’s where we feel the failure.” (formative focus group discussions)

At times, team members also felt burdened by having to decide which clients to offer resources to and which clients would not receive these resources. While these decisions were guided by policy, the team members felt implicated by their role in making these offers. One paramedic shared:

“I led a team which both offered hotel rooms to unhoused folks but also directed unhoused individuals to leave the area who weren’t eligible for shelter. I had to make arbitrary decisions on which individuals received a very coveted resource (hotel room) and which did not - despite some policies in place which helped guide the work, some challenging decisions and actions still had to be made. I wish we had more housing and shelter for folks so that it would not have to be triaged.” (moral injury assessment)

### Pathway 3: moral dilemmas can lead to moral distress for alternative response teams

Alternative response programs rely on being able to refer or transport clients to resources to meet their needs, like a sobering center or shelter. While the community members’ demands and insufficient resources can produce dilemmas, when these negatively impact clients or undermine the work that team members do to build trust with clients so that they can assist a person over the long term, this produces distress.

For example, when follow up care or connection to services did not happen reliably because of short staffing within referral partner agencies, this produced moral distress.

“We spend so much time talking to these people who are fearful of anybody in uniform. And then we make all these promises [and if the follow up team] doesn’t show up and meet them in the morning, then it’s like all that just went down the drain. We just looked like a bunch of liars.” (formative focus group discussions)

One paramedic leader reflected:

“[Team members] know it’s not right when they have to leave somebody on the street, in the rain, and they don’t have any options for them…. The biggest thing that bothers me is watching hospitals discharge people who should not be … they discharge them to the street. And we know that they have neurocognitive issues, we know that they’re at a high risk of death. And it’s hard to watch that. I know, it’s hard for the crews to watch that.” (formative focus group discussions)

Many people described the difficulty of working with clients with serious mental illness and substance use problems who decline services. One peer specialist team member said that it was “frustrating” when a resource is available but a client does not want to use it: “It’s raining outside. Like, why don’t you want to go inside, like, we have a shelter, right?” But the peer specialist also understood that “we may be offering them shelter but not knowing that the last time they were in a shelter they were assaulted, or their things were taken from them, or they were treated poorly by a staff member” (formative focus group discussion). In response to the moral injury assessment, several members shared about how encountering these dilemmas routinely produced distress:

“Seeing people who need assistance from a resource that we can’t access. For example, a person who is aggressive and denies resources. Or a person who I perceived as being so beaten down that they decline services which would help them. There are also many clients who have used the resources available, and do not want to use them in the future. I feel that far too many people are left on the streets, without resources to help them. I feel that the impact we do have is completely overshadowed.” (moral injury assessment)

Another participant wrote about the stress of witnessing “the slow burn of homeless. Seeing the same people who don’t want help and some who do but can’t get the help needed due to the broken system. Everyone takes energy. And the ones that don’t want help but cross our paths the most seem to take the most energy” (moral injury assessment). One respondent asked, “By maintaining their autonomy, are we perpetuating the problem or allowing them free will to live as they see fit?” (moral injury assessment). A peer support specialist said, “they need to do better to equip people in our position for what we are encountering” (peer support specialist advisory board meetings).

### Pathway 4: work conditions can cause moral distress, injury, and burnout for alternative response teams

There were many ways that working conditions contributed to moral distress, injury, and burnout. Compared to other forms of peer work, social work, and emergency medical services, the goals of alternative response programs and metrics of success are more contested. Many people are involved in developing these programs and different entities and agencies may have different goals for the program and different ways of measuring success. Goals may include shifting calls from law enforcement to the alternative response program, connecting clients to resources, reducing transportation to hospitals, or relieving strain on ambulances in order to shorten response time for medical emergencies. At times, these goals are incompatible with each other. For example, in order to relieve strain on ambulances, teams need to spend a limited amount of time on each call so they can get back into service and respond to the next behavioral health crisis call instead of having that call dispatched to an ambulance. However, in order to connect a client to a resource, the team may need to spend a lengthy time on a call, building rapport with the client and carefully ascertaining what resource the client may be interested in accepting.

One team member said, “I think part of the problem is the goal isn’t defined. Everyone’s goals on these things are different” (formative focus group discussion). Another team member described how responding to crises but not doing follow-up felt incompatible with their internalized sense of how to accomplish the work:

“One of my biggest, biggest frustrations with this [program] is that technically we aren’t allowed to follow up … It’s so important to have that [opportunity to build rapport], and yet I feel like we’re not technically allowed to build that. And that’s the key to the success” (formative focus group discussion).

In a context where the work is morally fraught, having a shared vision for the work and explicit communication about the indicators for success is particularly useful.

In addition, the alternative response program space is dynamic and policies that determine which calls team members are dispatched to or co-response protocols with law enforcement changed several times during study. Many team members expressed feeling “blindsided” by these changes and noted that they received no additional training on how to respond to new types of calls. For example, one team member found out during a shift that they would now be responding to juvenile calls (peer support specialist advisory board meetings).

There was also a challenge of working in an interdisciplinary and interprofessional team, where team members bring very different perspectives and skills to their work and lack a cohesive vocabulary. One peer specialist, for example, described how tiring it was to try to interpret to paramedic partners the emotional needs of clients with psychotic symptoms: “They’re like, ‘how did you even hear that? They’re just speaking gibberish.’” And described how this can produce tension with coworkers: “After we’re like fighting for the rights of this person, then we’re on the rig trying to repair and trust each other as a team again” (peer support specialist advisory board meetings). Debriefing after calls was helpful for strengthening communication. Facilitated program debriefing was a standard practice when the program first started, but after several months facilitated debriefing ended. One paramedic recalled that when the team stopped debriefing “our problems would occur within the crews because the social workers, the peers, the therapists, the various people we worked with, we all have different communication styles. And on an ambulance, you have to make it work, and if you don’t, you can just sit and be quiet. But … in this environment, it was very different. It’s hard to drive all day long with two other people who don’t want to talk” (formative focus group discussion).

Some team members also described experiencing a hostile work environment when they were partnered with a team member who was aggressive or who used racist or biased language:

“There’s some teammates over the years where I’m like, ‘Ah, I have to get ready for this shift.’ I have to mentally be ready for a comment or … just something inappropriate being said…. I can do all my self-care, or whatever version of making sure I’m good [to be ready for the work serving clients], but then I have to go an extra step of preparing to be perhaps in an environment that’s hostile.” (peer support specialist advisory board meetings)

The team member went on to observe that one of the contributors to moral injury was actually the “combination” of having to be in this hostile professional environment while simultaneously working to “provide space and safety for our clients and the people we’re supporting” (peer support specialist advisory board meetings).

In addition to varying goals, changing protocols, being unable to meet clients’ needs, and challenges with other team members, additional work conditions that were identified as contributing to moral distress, moral injury, and burnout were lack of communication from leadership to team members; a sense of disconnect between decision-makers’ understanding of the work and team members’ experiences of the work; and understaffing, difficulty with taking leave, and mandatory overtime requirements. Team members also experienced physical strain from the work and sometimes injury.

### Pathway 5: interrelationship between moral injury and burnout for alternative response teams

While members may experience either burnout or moral injury, these experiences frequently co-occur. In our conceptual model, we display these with bidirectional arrows, noting that experiencing one of the issues can also increase the likelihood of experiencing the other.

Team members described how much more “mentally taxing” alternative response was compared to other programs they have worked in (emergency medical services for EMTs and paramedics, other non-crisis peer support programs for peer support specialists). One paramedic said, “once you’re downtown, you’re just in it, you’re just in the water, just bathing in this human tragedy” (formative focus group discussions). Another shared: “I think for all of us, we just have that want and that need to help people, and we feel like if we can’t successfully do that, we take it personal and it eats away at us” (formative focus group discussions). One paramedic reflected that each call working in alternative response feels like “we’re either going to get verbally abused that we’re there, or they want something we don’t have, or we want to sell them something they don’t want … Maybe I should just pick up ambulance shift … [to] refresh my morale” (reflective focus group discussions).

The emotional exhaustion and reduced sense of professional efficacy experienced by team members was described as very different from what people experienced in other programs they have worked in as EMTs, paramedics, or peer support specialists.

“We’re put in a situation, and the public’s looking at us [to] help that person…. We’re put in this impossible role to fix this impossible situation that no one has an answer to anyway. And we’re all, you know, certificate holders. We’re not doctors. Not to downplay, but let’s call it what it is. Like, we are paramedics. We’re taught how to stop bleeds, and do CPR, and give a few medications, and stick tubes down people’s throats…. We’re not prepared for [this work].… We have all this pressure from all of these angles, and it’s like for me, that’s the moral injury. Where it’s like you’re all telling me to do this, but you all also acknowledge that there’s no solution for this. So what the? What do you want me to do?” (formative focus group discussions).

A peer specialist who had recently moved to a non-crisis peer response program after several years on the dispatched alternative response team reflected:

“I started street crisis and I stopped taking care of myself. I started street crisis and it was just like, I just wanted to go to work and go home and not do anything else. That work was so intense…. It was super triggering, super triggering….I don’t feel like I’m not able to do [peer] work because of that, I’m just feeling like [crisis peer work’s] not my avenue at this moment.” (peer support specialist advisory board meetings).

The persistent exposure to events where team members face ethical dilemmas, do not consistently have the resources to support clients, and do not feel they have the professional skills that are needed to meet the challenges they face set team members up for both moral injury and burnout. Once a person is already experiencing hallmark symptoms of burnout such as emotional exhaustion and reduced sense of professional efficacy, they may be more likely to have cognitions associated with moral injury. And once a person feels symptoms of moral injury like guilt, shame, and withdrawal, they are more likely to experience burnout.

### Feedback loop (Blue Pathway 6): witnessing inequities for clients produces moral distress for alternative response teams

An important aspect of our model is recognizing that alternative response teams respond to people who are already experiencing inequities and social marginalization. For example, many responders talked about getting to know clients who experienced severe sexual abuse as children, leading to homelessness, which led to substance use and mental health problems, leading to further victimization. Witnessing these situations and being unable to help produces moral distress. One paramedic described his devastation at the death of a client whom he had spent the last year trying to help: he felt as though the world had failed her.

In addition, the program operates within a system that itself produces and reinforces existing inequities. Team members described their distress at feeling complicit in the perpetration of these inequities. Team members identified groups of people more likely to be victimized on the street or in a shelter or for whom there were no resources available. For example, shelter policy required elderly clients to use bottom bunks and mobility impaired clients to use bottom bunks in a corner. This meant that elderly or disabled clients were less likely to be provided shelter than younger, non-disabled clients, leading to further inequity. Team members felt distressed by their role in perpetuating these inequities:

“You’ve got somebody in a wheelchair that’s homeless, or experiencing homelessness, they’re fucked. You can’t take them to a shelter. You have nowhere to take them. So you’re literally leaving somebody who’s handicapped in a wheelchair on the street. There’s shelters, but you can’t go there. What does that do to you morally, ethically? It’s, like, that is probably one of the worst things. Like, ‘oh, yeah, I get it. You’re in a wheelchair. Got nothing for you.’” (formative focus group discussions)

In addition, because this work can cause burnout, sometimes team members work with colleagues who are cynical and numb. When team members become cynical about their ability to connect clients to services, they may then spend less time on scene trying to find a resource that may benefit the client. For example, one peer specialist described their distress about an incident where they perceived their colleague to be too cynical and impatient with a client’s situation. The person they were dispatched to help was very upset about not being able to get a safe place to sleep. The peer reported that their colleague said the client was “too elevated” and “tried to clear the call … I was like, I’m not leaving. That’s crazy.” The peer specialist was able to find a shelter bed for the client that night where he could stay with his dog, but felt exhausted, distressed, and angry at not having support within the team (peer support specialist advisory board meetings).

Several concerns about fellow team members’ actions that could perpetuate inequities were described in the moral injury survey, which was the only data sources where people could share their experiences anonymously and privately. For example, one person wrote that they found it troubling when “I witnessed others in the field acting judgmentally toward patients and it affected the quality of care that was provided. Every patient, regardless of background or circumstances, is entitled to the same quality of care” (moral injury assessment). In our conceptual model, we recognize that adverse outcomes for team members can lead to adverse outcomes for clients. Witnessing these adverse outcomes for clients then further harms the first responders who are seeking to help them, perpetuating this negative feedback cycle.

### Strategies reported by alternative response team members to prevent and mitigate moral injury

Several people identified personal and organizational practices that helped them manage their distress that arose from these dilemmas during the focus groups, peer support specialist advisory board meetings, and the alternative response team peer conversations. These strategies included the following:

#### Centering around the purpose of the work and the philosophy of providing trauma-informed care

Providing trauma-informed care is about centering the client’s experience. One paramedic shared that rather than pushing a client, “[to] make a decision…. ‘Do you want to go to the hospital? Yes, no, I need a decision.’ Sitting on a scene, listening, letting somebody talk, that human element. I mean, that to me is you know, that’s as good as it gets for trauma informed care.” (formative focus group discussion). A peer support specialist shared a similar anchoring sentiment:

“In my head, I’m like, why don’t you want this?… We’re trying to get you into a place where there’s food, and there’s warmth, and there’s a shelter over your head. But that’s me being selfish to some degree, right, because that’s moving from a place where I’m no longer being client-centered because that’s not what they want at the moment, right?” (formative focus group discussion).

#### Centering around what is within the team member’s control and focusing on the limited scope within which they can contribute

For example, one paramedic shared this practice:

“A lot of the clients have been through trauma, various degrees of trauma, and they are standoffish … when I look at how my day went, and whether I was satisfied with the day itself, it’s not if the client went with me, or what kind of resources I got for them, but how did I present myself with them? Did I present myself in a way that is honorable that I can go home and say, ‘Yeah, I did the right thing’?” (formative focus group discussion).

This type of practice can be very difficult in the high-intensity crises that teams often respond to. One peer specialist described responding to a client who was “slamming his head onto things” experiencing a bad drug reaction. While she was trying to calm him and coordinate getting an ambulance crew to restrain him so he would not continue to injure himself, a bystander began screaming at her for participating in restraining the client, disparagingly calling her a “fucking cop.” She centered herself on the question “how do I be the healer in this moment?” And chose to hold the client’s head, continue to talk calmly to him, and try to shield others from the intensity of the moment: “I’m in front of this person who’s screaming. I’m going to hold his head” (peer support specialist advisory board meetings).

#### Noticing and appreciating when systems do work well

One team member reflected on a success he had just had:

“You’re defeated a lot of times. Like just calling that case manager that I just called. I was like… ‘This guy’s not going to answer the phone.’ And sure enough, he answered. [And he’s] like, ‘yeah, I’ll go to the hospital. I’ll go meet with him now…. We’re working on getting them conserved.’ And you’re so not used to that. I was, like, wow. All right. Cool. That’s, like, one golden egg. I mean, like, that rarely happens.” (reflective focus group discussion)

For this responder, noticing the success and then sharing those stories was a protective practice.

#### Relying on the support of colleagues to talk through difficult encounters

When the response program first began, the teams engaged in structured full team debriefings. While for paramedics and EMTs, in particular, debriefing calls is not part of their training, many found that when they collaborated with the social workers and peer specialists who routinely engaged in this practice, it was helpful:

“I think it was a little awkward for us … because it was a very unfamiliar process … But a few of us were quite surprised by how helpful that was”. (department report back)

Informal debriefing with team members was also highly valued:

“Everybody that I would go to for advice is here…. I never leave feeling that guilt, shame, anger that I used to on the ambulance because I know I talked to a buddy who understands it before I left and I feel better.” (formative focus group discussion)

#### Leaders support members with their own mental wellness

Like many behavioral health organizations with mental health providers, the peer specialists on the alternative response program have regular individual supervision where they can discuss challenging calls. In addition, as a behavioral health organization with a trauma-informed approach, leaders support members with their own mental health needs. For example, one peer specialist shared that after learning about someone they knew who had a fatal overdose, they had called their supervisor and said:

“I don’t think I can go to work for this shift. I need an extra day to process. But I also need somebody who can just listen to me and what I’m going through. And [my supervisor] acknowledged my experience … If I did not have a person to actually share that, I could actually get much more callous…. But after the conversation and the supervision and the support I got from [my supervisor], I felt much, much better and was ready for work.” (formative focus group discussion)

### Piloted and hypothesized interventions to reduce moral distress and prevent moral injury

Through the PAR study, we worked with team members and leadership to identify interventions hypothesized to prevent or mitigate moral injury, some of which we were able to pilot during the study. During formative focus group discussions and report back meetings, members brainstormed dozens of interventions that they thought could help reduce moral distress and prevent moral injury and burnout. These ranged from the aspirational (e.g. a month of paid vacation every three years) to the practical (e.g. cross-training with other partner agencies). **Table 3** maps examples of the interventions that were proposed by program members onto each of the pathways in the conceptual model to illustrate team members’ practice-informed suggestions to reduce moral distress and burnout and protect against moral injury.

**Table 3:** Example interventions proposed by team members and leadership to reduce moral distress, moral injury, and burnout mapped to conceptual model pathways.

Conceptual model pathway	Example interventions proposed by team members
Pathway 1: Broader community members’ demands can create moral dilemmas for alternative response teams	• Training on how to skillfully communicate with the public. • Develop a brochure that members can give to the public when they are responding to a crisis call. • Public education campaign to inform the public about the scope of work of the alternative response program and the way that they interface with other city agencies supporting the unhoused population.
Pathway 2: External resources and policies to support the client population can create moral dilemmas for alternative response teams.	• Communication from leadership about advocacy with city leadership to improve resources. • Develop data tracking system to capture when resources are not available to use for advocacy. • Cross-training with other agencies and referral partners to promote increased mutual understanding.
Pathway 3: Moral dilemmas can lead to moral distress for alternative response teams.	• Call debriefs, case reviews, formal workshopping of difficult calls and supervision. • Creating supportive spaces for shared discussion of morally complex situations. • Sharing featured success stories.
Pathway 4: Work conditions can cause moral distress, injury, and burnout for alternative response teams.	• Communal meals while on shift and other casual teambuilding time. • Working with leadership and members to recognize signs and symptoms of moral injury and burnout. • Interprofessional communication training to understand the different perspectives, skills, and training that each team member brings to the program.
Pathway 5: Interrelationship between moral injury and burnout for alternative response teams.	• Group therapy, peer support groups, individual therapy and/or psychoeducation on site. • Opportunity to exercise during shift if call volume allows, similar to other Department members. • Develop sabbatical program or opportunity to transfer briefly to a different response program. • End mandatory overtime practices.

During the course of the PAR, we piloted some of the practice-informed suggestions for intervention and worked with leadership to support implementation of additional strategies to reduce moral distress, moral injury, and burnout. One of our key strategies was to acknowledge the moral and ethical challenges of the work and to create space for team members to talk with each other about these dimensions. This built on an observation that during the formative focus groups, team members valued the opportunity to be in dialogue with one another:

“listening to [my colleague] describe perfectly what I think … there was something healing about that … I didn’t know he felt that exact way … I’m glad I’m not alone in this.” (formative focus group discussion)

The piloted interventions focused on creating both structured and unstructured spaces and times to build this routine acknowledgement. During the facilitated peer conversations, we introduced language and explanatory frameworks to help team members reflect on their experiences and then held space for dialogue. As one paramedic reflected, these facilitated sessions helped with “putting a name to what it was that we’ve been looking at and witnessing. It’s sort of like these are things we recognize, these are there” (reflective focus group discussion). Learning frameworks to better understand the client population and the impact of stressors and moral injury on first responders was “confirming and validating … it provides an actual name for the feelings you’ve had” (reflective focus group discussion). One team member shared, “it helps to feel more empowered in your job when you know more information, you have more power, you have more knowledge to be able to execute what it is that we’re supposed to be doing out there” (reflective focus group discussions).

These conversations then continued into unstructured spaces, including weekly meals that teams could take together on shift. The meals provided an opportunity to connect with colleagues, share experiences, exchange ideas, and decompress. Because of the conversations that happened in facilitated spaces, relationships between colleagues also changed. In one reflective focus group, a participant shared:

“I like the team dynamic of it, getting to know my coworkers deeply…. [It’s] different being able to come together and have a conversation with one another within an open forum … Now I feel like I can turn to somebody and actually have a conversation. Like [my colleague] and I have deep conversations, actually, all the fucking time [now] … I don’t think him and I have ever connected [before] … We’ve known each other a long time … but having a foundation of being able to be with one another. I think it’s going to be a like, for long-term success and like stress mitigation, I think this is like where we start.” (reflective focus group discussion)

In addition to having new language and deeper connections, members really appreciated the opportunity to reflect on the moral dilemmas that they experienced through their work with a deliberate invitation to talk with colleagues about the ethical dimensions of what they witnessed. For example, one person brought up a client whom they had known for the years:

“I’m thinking people like our guy with the leg wounds that were there forever…. He was alert and oriented, so he doesn’t really meet the 5150 criteria, but we literally watched his legs disintegrate to the point that they were going to amputate. And he ended up so far down that road that he ended up losing his life to it ultimately. This raises the moral conflict of, like, do you allow somebody to continue down the road on their own free will when they’re using substances? I don’t know…. No matter what you do, you’re going to be wrong. You leave them in the community, you’re wrong. You take them [to the hospital], you’re wrong. And, so there’s all these things that you’re trying to make the right decision.” (reflective focus group discussion)

Another participant reflected, “Even though you know you’re kind of doing the right thing for that person and the community, it still doesn’t feel good … It’s complicated work. It is the most complicated job I’ve had” (reflective focus group discussion). These sessions were so positively received that Fire Department leadership institutionalized them for the alternative response teams by contracting with the employee assistance program to provide a therapist to facilitate biweekly peer conversations on an ongoing basis.

An additional thrust of the interventions was to improve communication internally within the program, externally with partners like shelters and law enforcement agencies, and externally with the community. One illustration of this strategy is around communication with law enforcement during times that they co-respond to a client. Conflicting or vague policies dictating how to respond to a person in crisis can put alternative response team members at risk of physical harm (e.g. police policy on disengagement aimed at de-escalation to mitigate risk of harm may mean that alternative response team members need to physically subdue a client who is acting violently if that person is unarmed). After an incident where team members were injured by a client while police stood by, program leadership met with police leadership to carefully review the case and create a shared document on how different first responders should engage with each other on scene, which they laminated and gave to all alternative response team members. One of the program leaders reflected that witnessing their team be injured in this incident had been a source of moral distress for them as a leader. They were able to turn that distress into an action they could take that would help reduce the chance that this situation would be repeated. Team members appreciated this responsive and proactive strategy and the communication they received about the leader’s actions.

## Discussion

Engaging in participatory action research allowed us to collaborate with those involved in the work to understand that moral distress and moral injury are critical issues for alternative response teams. Through a variety of modalities and conversations, participants were able to describe their exposures to PMIEs and the impact that ongoing exposure has on their wellbeing. Participants also shared strategies for coping and potential interventions, some of which could be implemented during the study period due to the iterative PAR approach. The conceptual model that we share here was developed through many months of listening as participants shared their experiences, impacts, and solutions. Indeed, the PAR approach allowed for continuous member checking and participant validation.

While we were able to draw to some extent from existing models, we recognized that there were unique exposures for alternative response teams that differed from other groups both due to the quality and quantity of their daily interactions. Participants faced these exposures daily, working with the most vulnerable and underserved populations. While many existing models have been developed with war veterans, these models may be limited in their applicability to alternative responders; typically, by the time veterans seek treatment, they have come home from the war zone. Alternative response teams continue their exposure, often on a daily basis, and it is a part of the job, with little relief. Consequently, models like Litz et al. (2) are limited due to the sole focus on the intrapersonal development of moral injury, rather than a multidimensional model that takes societal, structural, and interpersonal factors into account.

Griffin et al.’s (30) Dimensional Contextual Model of Moral Injury, focusing on healthcare workers, expands the Litz et al. (2) event-specific model by including individual level, interpersonal, organizational, and societal factors that impact moral injury. Importantly, alternative responders in our study reported multiple exposures at each of these levels, demonstrating that any model applied to this group needs to be multidimensional to fully account for these exposures and their impacts. However, this model does not necessarily account for how organizational or societal levels produce PMIEs, an important part of moral injury risk for alternative response teams, who as part of their work are continuously interfacing with the community, partners, and leadership, each with different sets of expectations. Our participants shared that working with vulnerable populations with multifaceted needs can be a precursor for moral injury when societal or organizational demands are not in line with possible solutions due to lack of resources, policies, or other barriers. Consequently, our model expands Grifffin et al.’s (30) framework by highlighting the societal and organizational forces that can contribute to creating moral injury.

Our model also builds on Linzer and Poplau’s (33) Conceptual Model for Moral Injury and Burnout Prevention, which recognizes the unique aspects of moral injury and burnout and also demonstrates how they are closely tied and need to be conceptualized together when thinking about healing and intervention. Their model is the basis for the pathway that describes moral dilemmas contributing to moral distress, which can produce moral injury, which is in turn bidirectionally related to burnout. While their model focuses on factors in the workplace, we have expanded the model by including the societal/political drivers and determinants, as well as sources of exposure to moral dilemmas that contribute to distress. We also have added explicit adverse outcomes for both first responders and vulnerable clients with whom they work, which is a unique piece as well. We learned that adverse outcomes experienced by vulnerable populations and witnessing inequities can also be part of the cycle that creates risk for moral injury and burnout among first responders.

Our model is also compatible with the National Framework for Addressing Burnout and Moral Injury in the Health and Public Safety Workforce, and specifically what the model describes as “relational drivers” (34). Consistent with this framework, we found that strategies to reduce moral injury and burnout need to focus not just on the individual level (e.g. mindfulness training), but also on operational and relational drivers of burnout and moral injury that occur interpersonally within the workplace. This is also consistent with the call to focus on “systemic factors” in the prevention of moral injury in healthcare workers (35).

We have built the Alternative Response Program Moral Injury and Burnout Model to reflect the voices and experiences of first responders and to expand existing models of moral injury in high-risk occupations. We have also described interventions at the individual and organizational level that were helpful in the context of the alternative response program in the PAR and reflect team members’ suggestions and preferences. To achieve these goals, we were able to leverage the strength of PAR approach, the simultaneity of research to understand and efforts to intervene on the identified problems. The utility of these interventions can serve as further evidence of the validity of the interpretation of the problem and its causes.

There were also requests for changes at the level of the city or broader society that first responders believed would also reduce their exposure to potentially morally injurious events and to moral distress and injury, but were outside the scope of our study to pilot. These changes included better coordination of services to support unhoused individuals so clients did not fall through cracks in the system and more housing resources, sobering or detox centers, and specific targeted resources like inpatient psychiatric beds to help first responders meet their client’s needs.

The majority of interventions thus far that focus on moral injury have been developed for veterans of war and focus on ameliorating mental health symptoms at the individual level. While this approach can be incredibly beneficial for individuals suffering with functional impairment and moral injury symptoms, there is often a limited focus on the interpersonal, organizational and societal contributors of moral injury. This is particularly challenging for individuals in high-risk occupations that continue to be exposed to PMIEs as part of their work. This model and insights gleaned from the PAR approach demonstrate how important it is to think systemically, organizationally, and interpersonally, about interventions. The PAR approach can be used with high-risk occupational groups to develop workplace and system-wide interventions with alternative response teams and other high-risk groups. We found that when creating a supportive space to brainstorm, members were readily able to identify interventions they thought would reduce their moral distress, moral injury and burnout. Additionally, being able to pilot test interventions within and across systems based on the model contributed to a deeper understanding of how to make an impact with this population. Consequently, the Moral Injury and Burnout model can guide how we think about interventions at multiple levels.

Several limitations should be noted when interpreting our findings. This model was developed through close collaboration with one alternative response program as a starting point and should not be generalized to other alternative response programs without a deeper understanding of the work context and organizational challenges. More specifically, the landscape of alternative response programs is diverse and rapidly changing and so this model may need to be expanded or modified to capture the diverse exposures, contexts, and experiences of other alternative response program members.

Although qualitative work is not meant to generalizable, future work can build on experiences of similar at-risk occupational groups. While our intention with these qualitative illustrations is to provide exemplars, and we recognize that each of these may not arise for all team members in all programs. Through conversations with leaders and members in other alternative response programs and with first responders who frequently respond to community members experiencing homelessness, addiction, and mental health challenges outside of specialized programs, we believe it is likely that the framework for the model is applicable to other programs. Specifically, the model depicts sources of moral dilemmas as systemic pressures, and work conditions as mediating movement on a continuum between normative stress through moral distress and moral injury, and burnout. While the specific systemic pressures and work conditions are context-dependent, we posit that this model is a good starting point to understand and pilot test the best interventions for any specific group. In keeping with the PAR approach, we recommend leaders and multidisciplinary members in alternative response programs work together to adapt this model to their specific contexts and identify the interventions most likely to prevent the development of moral injury and burnout within their program.

Given that moral injury and burnout are growing problems in alternative response programs, our goal was to amplify voices of this group to highlight both their struggles as well as proposed solutions and interventions. By working closely with this community, we were able to develop a model that resonates for these first responders and can hopefully help guide a better understanding of issues and proposed pathways for interventions among alternative responders and other high-risk groups.

## Data Availability

The datasets presented in this article are not readily available because there is a risk of re-identification even of anonymized data. Requests to access the datasets should be directed to MW, miranda.worthen@sjsu.edu.
